# Heterogenic Distribution of Aromatic L-Amino Acid Decarboxylase Neurons in the Rat Spinal Cord

**DOI:** 10.3389/fnint.2017.00031

**Published:** 2017-11-08

**Authors:** Li-Qun Ren, Meng Chen, Hans Hultborn, Sen Guo, Yifan Zhang, Mengliang Zhang

**Affiliations:** ^1^Department of Neuroscience, University of Copenhagen, Copenhagen, Denmark; ^2^Laboratory of Spinal Injury and Rehabilitation, Chengde Medical College, Chengde, China; ^3^Neuronano Research Center, Department of Experimental Medical Sciences, Lund University, Lund, Sweden

**Keywords:** monoamine, neurotransmitter, interneuron, non-monoaminergic cell, monoenzymatic cell, D-cell, CSF-contacting cell

## Abstract

Aromatic L-amino acid decarboxylase (AADC) is an essential enzyme in the synthesis of serotonin, dopamine, and certain trace amines and is present in a variety of organs including the brain and spinal cord. It is previously reported that in mammalian spinal cord AADC cells (called D-cells) were largely confined to a region around the central canal and that they do not produce monoamines. To date, there has not been a detailed description of their distribution and morphology in mammals. In the present study this issue is systematically investigated using immunohistochemistry. We have found that AADC cells in the rat spinal cord are both more numerous and more widely distributed than previously reported. In the gray matter, AADC neurons immunolabeled for NeuN were not only found in the region around the central canal but also in the dorsal horn, intermediate zone, and ventral horn. In the white matter a large number of glial cells were AADC-immunopositive in different spinal segments and the vast majority of these cells expressed oligodendrocyte and radial glial phenotypes. Additionally, a small number of AADC neurons labeled for NeuN were found in the white matter along the ventral median fissure. The shapes and sizes of AADC neurons varied according to their location. For example, throughout cervical and lumbar segments AADC neurons in the intermediate zone and ventral horn tended to be rather large and weakly immunolabeled, whereas those in comparable regions of sacrocaudal segments were smaller and more densely immunolabeled. The diverse morphological characteristics of the AADC cells suggests that they could be further divided into several subtypes. These results indicate that AADC cells are heterogeneously distributed in the rat spinal cord and they may exert different functions in different physiological and pathological situations.

## Introduction

Aromatic L-amino acid decarboxylase (AADC) is an essential enzyme in the conversion of 5-hydroxytryptophan (5-HTP) to 5-hydroxytryptamine (5-HT, serotonin) and L-dihydroxyphenylalanine (L-dopa) to dopamine (DA) (Lovenberg et al., 1962; Christenson et al., 1972). This enzyme is also involved in the synthesis of trace amines such as tyramine from tyrosine, 2-phenylethylamine from phenylalanine, and tryptamine from tryptophan (see reviews by Zhu and Juorio, 1995; Berry, 2004; Gozal et al., 2014). AADC has been found in many different organs including kidney, liver, lungs, blood vessels, brain, and spinal cord in many animal species as well as humans (Holtz et al., 1938; Hökfelt et al., 1973; Hardebo et al., 1979; Jaeger et al., 1983, 1984; Zhu and Juorio, 1995; Kubovcakova et al., 2004; Kitahama et al., 2009). In the brain AADC has been found to be expressed in multiple regions from the olfactory bulb to medulla oblongata (Hökfelt et al., 1973; Jaeger et al., 1984; Mura et al., 1995; Kitahama et al., 2009; Ugrumov, 2009; Blechingberg et al., 2010; Ahmed et al., 2012). Generally AADC neurons in the brain can be divided into two classes: monoaminergic (also called bienzymatic) and non-monoaminergic (also called monoenzymatic) neurons. The former can be further divided into DA-producing and 5-HT-producing neurons depending on whether the neurons also contain tyrosine hydroxylase (TH) or tryptophan hydroxylase (TPH) (Hökfelt et al., 1973; Tison et al., 1991; Ugrumov, 2009). The cells containing only AADC, but neither TH nor TPH, are also called D-cells (Jaeger et al., 1984). D-cells exist in many CNS regions from the spinal cord to the rostral forebrain in mammals. In the spinal cord AADC-only cells are called D1-cells, whereas those in other brain regions are designated D2 to D14-cells according to their locations from caudal to rostral (Jaeger et al., 1983, 1984).

Many earlier studies have demonstrated that following spinal cord injury (SCI) 5-HTP or dopa can be actively converted to 5-HT or DA (Viala and Buser, 1971; Bedard et al., 1979; Chandler et al., 1984; Hayashi et al., 2010). Although there were no morphological data from these studies confirming that the monoamines were produced in the spinal AADC cells, recent findings including those from our group (Li et al., 2014; Wienecke et al., 2014; Azam et al., 2015; Zhang, 2015; Hou et al., 2016; Ren et al., 2016) suggest that this is the case. Using a sacral 2 (S2) spinal transection rat model (Bennett et al., 1999), we (Wienecke et al., 2014; Ren et al., 2016) and Li et al. (2014) have demonstrated that AADC cells below the lesion increase their ability to utilize exogenous 5-HTP or L-dopa to synthesize 5-HT or DA, which could in turn increase motoneuron excitability. These results indicate that AADC cells may serve as a kind of reserve cells playing a critical role in compensating for lost monoamine innervations from the brain, and thus may be important also for motor functional recovery in SCI, although the overwhelming expression of AADC in capillary pericytes impairs capillary blood flow and thus motor function (Li et al., 2017).

Although AADC cells in the mammalian spinal cord have been reported in the region around the central canal (Jaeger et al., 1983, 1984), there has been no systematic investigation of their distribution. Recently, Li et al. (2014) claimed that following S2 transection in rats a novel group of neurons, which does not exist in healthy animals, appears in the lateral part of the sacrocaudal (S + Ca) spinal cord. However, we found that AADC cells are widely distributed in several different regions in the S + Ca spinal cord both in spinalized and normal rats and there is no apparent difference between these preparations with respect to cell distribution and numbers (Wienecke et al., 2014). Thus, it is important to make a systematic investigation of AADC cell distribution in the entire spinal cord. The preliminary results have been reported previously in abstract form (Zhang et al., 2012).

## Materials and Methods

### Animals and Tissue Preparations

All experiments were conducted in accordance with the guidelines of the *EU Directive 2010/63/EU* and were approved by the Danish Animal Experiments Inspectorate and the Malmö/Lund Animal Ethics Committee on Animal Experiments. All efforts were made to minimize the number of animals used and their suffering. In total, 12 normal adult male Wistar rats were used with a body weight of 150–250 g. The rats were transcardially perfused with 4% paraformaldehyde in 0.1 M phosphate buffer. The brain and the entire spinal cord were removed immediately and the latter was further separated into cervical (C), thoracic (T), lumbar (L), and S + Ca segments and post-fixed in the same fixative for 2–24 h at 4°C. Following post-fixation the brain and spinal cord were cryoprotected in 0.01 M phosphate-buffered saline (PBS) with 30% sucrose for up to 48 h at 4°C. In five rats the spinal tissue from C1 to S4 was further divided into single spinal segments and then cut transversely, and the part from Ca1 to Ca3 was cut horizontally into 40-μm-thick sections with a sliding microtome. In another seven rats the spinal segments from C1-8, T1-6, T7-13, L1-6, and S + Ca were cut either horizontally (five rats) or parasagittally (two rats) into 40-μm-thick sections. To test the specificity of AADC antibodies a brainstem was cut transversely into 40 μm sections. The sections were either immediately processed for immunohistochemistry or put into PBS with 30% sucrose and kept frozen at -80°C until used. In addition, some sacral spinal sections from a spinalized rat (70 days post-injury) stained with an AADC antibody and used in our previous study (Wienecke et al., 2014) were also included in the present report.

### Antibody Characterization

Two AADC antibodies were used to localize AADC cells in the rat spinal cord. Additionally, a number of other antibodies were used for double immunohistochemistry. An antibody was chosen based on the criteria that its specificity had been validated in our previous studies, by other scientists and/or the manufacturer. Omission control was routinely performed for all the antibodies.

**FIGURE 1:**
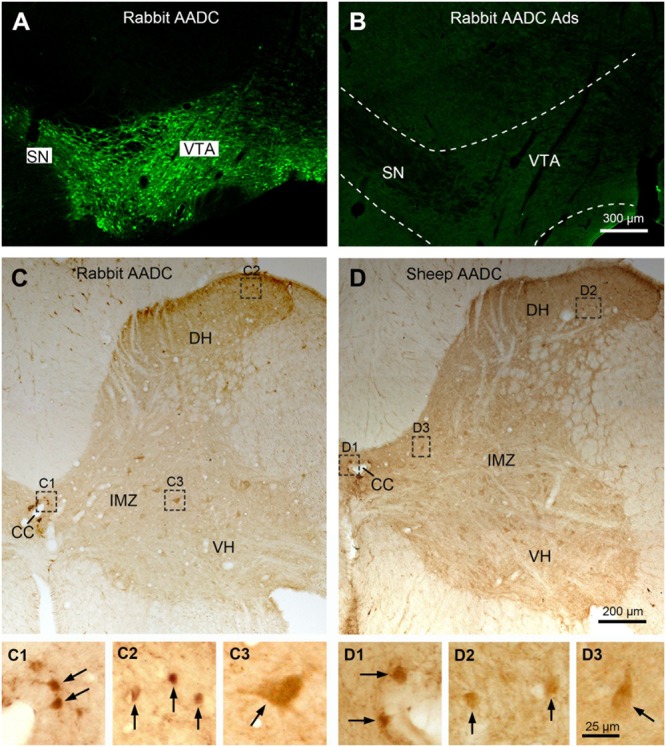
Specificity control of Aromatic L-amino acid decarboxylase (AADC) antibodies. **(A)** Clearly immunolabeled cell bodies were seen in the substantia nigra (SN) and ventral tegmental area (VTA) in the midbrain when the sections were immunostained with rabbit AADC antibody. **(B)** When control staining was performed with the antibody pre-adsorbed (Ads) with whole long AADC recombinant proteins the specific AADC immunolabeling became absent in the midbrain. **(C,D**) Rabbit and sheep AADC antibodies produced a similar immnuolabeling pattern in the spinal cord. **(C,D)** were from adjacent sections from C6 segment. **(C1–C3)** and **(D1–D3)** are enlargements of the areas demarcated with squares/rectangles in **(C,D)** showing the details of the AADC-labeled neurons (arrows) around the central canal (CC), in the dorsal horn (DH), and in the intermediate zone (IMZ). VH, ventral horn. Scale bar in **(B)**, valid for **(A,B)**, 300 μm; in **(D)**, valid for **(C),(D)**, 200 μm; in **(D3)**, valid for **(C1–D3)**, 25 μm.

In the present study the AADC antibodies used were sheep anti-AADC (AB119) and rabbit anti-AADC (AB1569) antibodies, both from Merck-Millipore. The antibodies were produced by immunizing the animals with recombinant bovine AADC proteins expressed in *Escherichia coli* and purified of inclusion bodies (according to company data sheets). The specificity of the sheep anti-ADDC antibody has been validated by western blot and adsorption experiments in our previous study (Wienecke et al., 2014). The rabbit anti-AADC antibody has been widely used to detect AADC immunoreactivity in different structures of the brain in different species (e.g., Ahmed et al., 2012; Stansley and Yamamoto, 2013). In addition, control immunohistochemical staining was performed using the same procedures on spinal cord and/or brainstem sections with the primary antibodies omitted or pre-adsorbed with whole long AADC recombinant proteins of human origin (200 μg/ml; Cat. No: NBC1-25854, Novus), with no detectable specific staining (**Figure 1**).

### Single Immunohistochemistry

To examine the distribution of AADC immunoreactivity in the rat spinal cord avidin–biotin complex (ABC) peroxidase immunohistochemistry was performed with either rabbit or sheep anti-AADC antibody. Spinal sections from five rats whose spinal cords were cut transversely were processed with rabbit anti-AADC or sheep anti-AADC antibody. In addition, spinal sections from three rats whose spinal cord were cut horizontally were processed with rabbit anti-AADC antibody. Spinal sections from two rats whose spinal cord were cut horizontally and from one rat whose spinal cord was cut parasagittally were processed with sheep anti-AADC antibody. For the spinal cords cut transversely 1 out of 10 sections from spinal segments C1 to S4 was processed, and for the spinal cords cut horizontally or parasagittally all the sections from different segments were processed. The detailed immunohistochemical procedure has been described previously (Wienecke et al., 2014). In brief, the sections were first incubated in either rabbit anti-AADC (1:2000–3000) or sheep anti-AADC (1:400) primary antibody for 40–48 h at 4°C and then in biotinylated goat anti-rabbit IgG (1:1000, Dako) or donkey anti-sheep IgG (1:200, Abcam) for 1 h at room temperature. After incubating in ABC (1:100, Vector Laboratories) the reaction was visualized in 0.05% diaminobenzidine tetrahydrochloride (Sigma–Aldrich) with 0.005% H_2_O_2_. Sections were thoroughly washed with PBS or Tris buffer between different steps.

### Double Immunohistochemistry

To investigate the phenotypic properties of the AADC cells in the spinal cord, double immunohistochemistry was performed in selected transverse (from four rats) or parasagittal (from one rat) sections from different parts of the spinal cord. Either rabbit or sheep anti-AADC antibody was used depending on the species from which the paired antibody was generated. To examine whether AADC is expressed in mature neurons, rabbit anti-AADC (1:500) and mouse anti-neuronal nuclei (NeuN, 1:500; Cat. No.: MAB377, Merck-Millipore) antibodies were used; to examine whether AADC is expressed in immature neurons, sheep anti-AADC (1:200) and rabbit anti-doublecortin (1:1000; Cat. No.: ab18723, Abcam) antibodies were used; to examine whether AADC is expressed in cholinergic neurons, rabbit anti-AADC (1:500) and goat anti-choline acetyltransferase (ChAT, 1:100; Cat. No.: AB144P, Merck-Millipore) or goat anti-vesicular acetylcholine transporter (VAChT, 1:250; Cat. No.: ABN100, Merck-Millipore) antibodies were used; to examine whether AADC is expressed in glial cells, either rabbit anti-AADC antibody (1:500) was combined with mouse anti-glial fibrillary acidic protein (GFAP, an astrocyte marker, 1:500; Cat. No.: MAB360, Merck-Millipore), mouse anti-adenomatous polyposis coli (APC, an oligodendrocyte marker, 1:100; Cat. No.: OP80, Merck-Millipore) or goat anti-ionized calcium-binding adaptor molecule 1 (Iba1, a microglial marker, 1:100; Cat. No.: ab5076, Abcam) antibody, or sheep anti-AADC antibody (1:200) was combined with rabbit anti-brain lipid-binding protein (BLBP, a radial glia marker, 1:1000; Cat. No.: ab32423, Abcam) antibody. The detailed double immunofluorescent staining protocol has been described previously (Kong et al., 2011; Ren et al., 2013; Wienecke et al., 2014). In brief, the sections were first incubated in paired primary antibodies for 24–48 h at 4°C and subsequently incubated in corresponding paired fluorescent secondary antibodies, which included donkey anti-rabbit Alexa Fluor 594 (1:200), donkey anti-rabbit Alexa Fluor 488 (1:200), donkey anti-sheep Alexa Fluor 488 (1:100–200), donkey anti-goat Alexa Fluor 594 (1:200), and donkey anti-mouse Alexa Fluor 594 (1:200). Secondary antibody combinations were chosen so that the AADC labeling always was green and the other labeling red. All the fluorescent secondary antibodies were from Invitrogen.

### Data Analysis

The spinal sections were observed with a conventional light microscope (Leica DM6000B, Leica Microsystems). Images were captured digitally and processed with Adobe Photoshop CS5 or CS6. For quantitative analysis of AADC-immunoreactive (IR) cells the MD Plotting System (AccuStage) was used. AADC cells were plotted in three rats whose spinal cords were cut transversely (except the caudal part which was cut horizontally) and processed with rabbit anti-AADC antibody and the ABC method. In these three rats every immunostained section from C to Ca segments was plotted. Different symbols were used to represent AADC-IR cells in relation to their different locations and sizes, and the numbers of the cells in different subsets were calculated with the program provided with MD Plotting System. Tissue volumes were calculated from the surface area and thickness of the plotted sections. Finally, the number of AADC cells was expressed per cubic millimeter spinal tissue. For the reasons stated in the Section “Results,” the sections processed with sheep anti-AADC antibody were not plotted. As a small number of AADC cells were found to be sparsely distributed in the ventral funiculus along the ventral median fissure, these cells were counted manually in all the sections from three rats whose spinal cords were cut horizontally and processed with rabbit AADC antibody. Their density was expressed as cell number per spinal segment.

Statistical analysis was performed using one-way ANOVA or one-way ANOVA on Ranks followed by Tukey’s test with SigmaPlot (version 11.0, Systat Software). The significance level was set as *P* < 0.05. The average group value was expressed as mean ± standard deviation (SD).

## Results

### AADC Antibody Specificity Controls

Two different AADC antibodies were used in this study: rabbit and sheep anti-AADC antibody. The specificity of the sheep antibody has been validated previously (Wienecke et al., 2014). In the present study we have performed adsorption experiments for rabbit anti-AADC antibody with the antibody pre-adsorbed with whole long AADC recombinant proteins of human origin and the AADC immunolabeling was completely abolished in the sections from the brainstem (**Figures 1A,B**) and the spinal cord (data not shown). No specific immunolabeling was detected when the primary antibodies were omitted (data not shown). These two AADC antibodies produced a generally similar immunolabeling pattern in the rat spinal cord (**Figures 1C,D**), that is, AADC-IR cells were detected around the central canal, in the dorsal horn, the intermediate zone, and the ventral horn although, especially in the dorsal horn, the sheep antibody usually labeled fewer AADC cells than rabbit antibody. Therefore, the description of the general distribution of AADC cells in the spinal cord is mainly based on the results obtained using rabbit AADC antibody.

### General Distribution Pattern of AADC-IR Cells in the Spinal Cord

**FIGURE 2:**
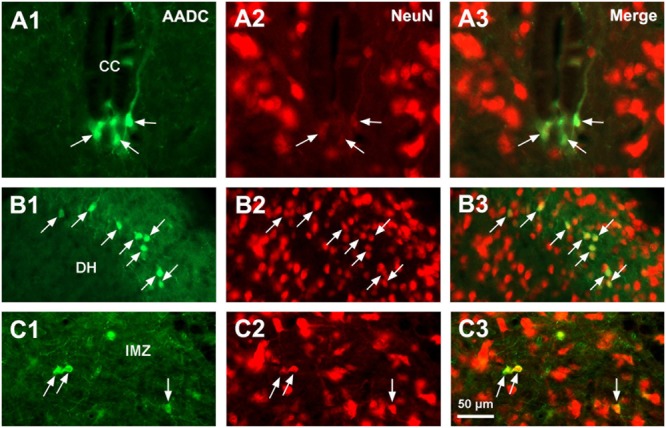
Aromatic L-amino acid decarboxylase and NeuN double-immunostaining indicates that AADC cells in the spinal gray matter are neurons. **(A1–C3)** AADC neurons around the CC were weakly immunolabeled with NeuN **(A1–A3)**, whereas in the DH **(B1–B3)** and the IMZ **(C1–C3)** they were densely immunolabeled with NeuN (arrows). AADC antibody was from rabbit. Scale bar in **(C3)**, valid for all panels, 50 μm.

**FIGURE 3:**
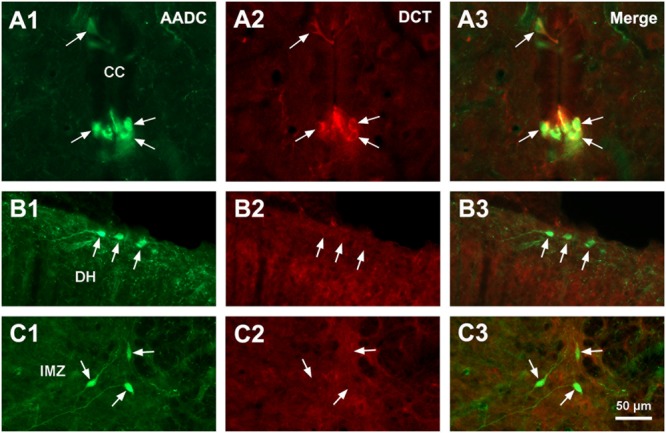
Aromatic L-amino acid decarboxylase and doublecortin (DCT) double-immunostaining indicates that AADC cells in different parts of the spinal gray matter are in different states of maturity. **(A1–C3)** AADC neurons around the CC were immunopositive for DCT **(A1–A3)**, whereas in the DH **(B1–B3)** and the IMZ **(C1–C3)** they were DCT-immunonegative (arrows). All images were from transverse sections at sacral segments. AADC antibody was from sheep. Scale bar in **(C3)**, valid for all panels, 50 μm.

**FIGURE 4:**
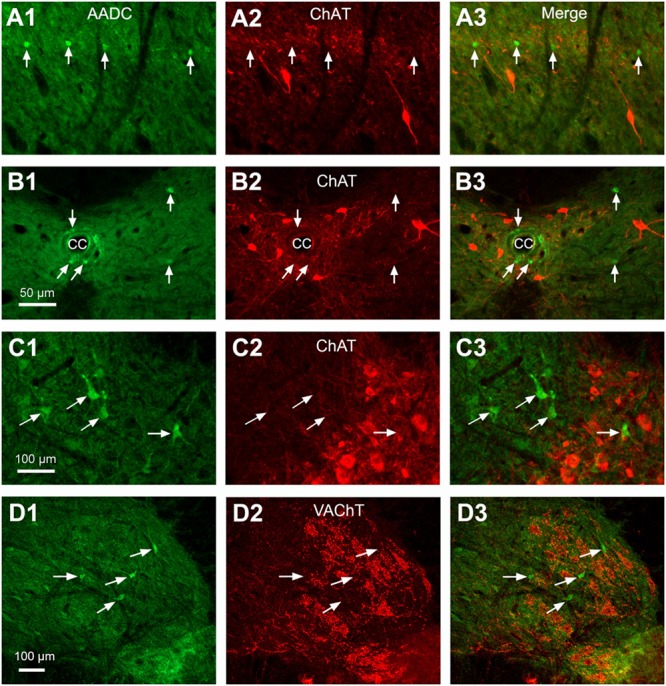
Double-immunostaining indicates that AADC neurons did not express ChAT or VAChT in different parts of the spinal gray matter. **(A1–A3)** In the DH. **(B1–B3)** Around the CC. **(C1–C3)** In the intermediate zone/ventral horn. In **(C3)** a ChAT-immunonegative AADC cell was located in a cluster of ChAT-immunoreactive (IR) motoneurons. **(D1–D3)** In the ventral horn VAChT-IR neurons did not express AADC. All images were from transverse sections at lumbar (L) segments. Arrows in different panels indicate AADC-IR cells. Cells colored red are ChAT- or VAChT-IR cells. AADC antibody was from rabbit. Scale bar in **(B1)**, valid for **(A1–B3)**, 50 μm; in **(C1)**, valid for **(C1–C3)**, and in **(D1)**, valid for **(D1–D3)**, 100 μm.

**FIGURE 5:**
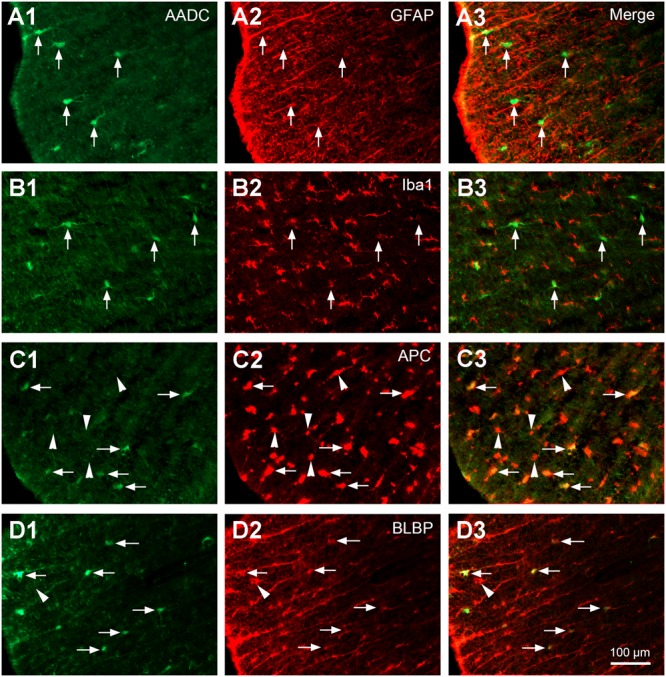
Aromatic L-amino acid decarboxylase cells in the white matter expressed oligodendrocyte and radial glial phenotypes but not astroglial or microglial phenotypes. **(A1–A3)** AADC was not expressed in astrocytes immunolabeled with GFAP (arrows). **(B1–B3)** AADC was not expressed in microglia immunolabeled with Iba1 (arrows). **(C1–C3)** AADC was expressed in a small proportion of oligodendrocytes immunolabeled with APC (arrows). Note that only the larger, but not the smaller, APC immunopositive cells (arrowheads) were double-labeled with AADC. **(D1–D3)** AADC was expressed in radial glial cells immunolabeled with BLBP (arrows). Still not all BLBP-immunolabeled cells were AADC-IR (e.g., marked by an arrowhead). AADC antibody was from rabbit for **(A1–C3)**, from sheep for **(D1–D3)**. All the images were from transverse sections of thoracic (T) segments. Scale bar in **(D3)**, valid for all panels, 100 μm.

**FIGURE 6:**
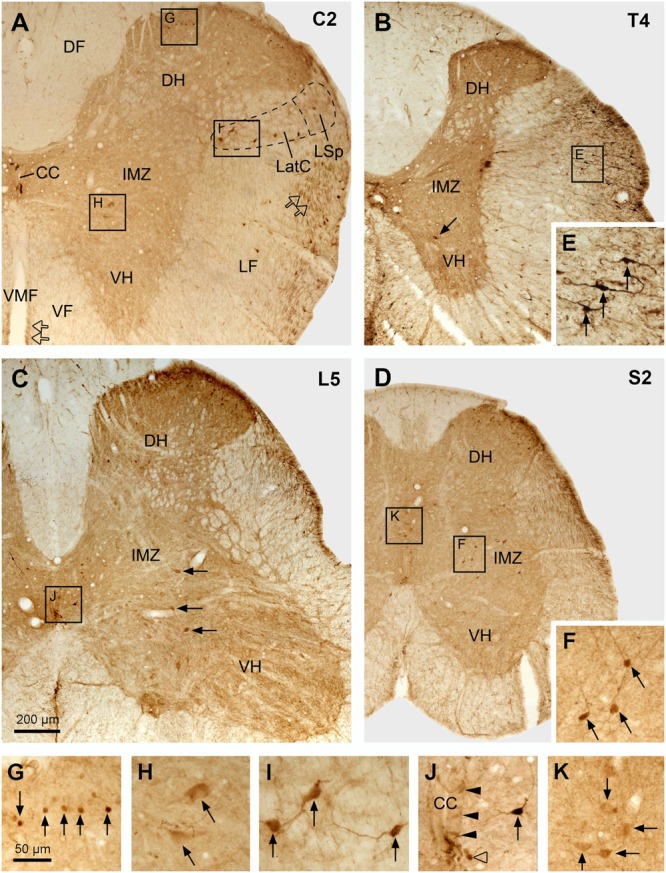
Aromatic L-amino acid decarboxylase cell distribution in different parts of the spinal cord illustrated in transverse sections. **(A–D)** Photomicrographs of low magnification from selected sections from cervical (C) **(A)**, thoracic (T) **(B)**, lumbar (L) **(C)**, and sacral (S) **(D)** segments. The spinal segment of the section is indicated in the upper right corner in each panel. Arrows in **(B,C)** indicate the larger neurons in the IMZ/VH in the T and L segment, respectively. Hollow arrows in **(A)** indicate AADC fibers in the lateral funiculus (LF) and ventral funiculus (VF) close to the ventral median fissure (VMF). CC, central canal; DF, dorsal funiculus; DH, dorsal horn; LatC, lateral cervical nucleus; LSp, lateral spinal nucleus. **(E–K)** Enlargements of the areas in **(A–D)** demarcated with the squares/rectangles showing the details of the AADC-labeled neurons (arrows or arrowheads) in the white matter **(E)**, superficial DH **(G)**, IMZ (larger cells in **H** and smaller cells in **F**), LatC **(I)**, around the CC (**J**, arrowheads indicate the AADC cells around the CC, arrow indicates a AADC cell slightly further away from the CC), and the deep DH (**K**, here the cells were from the sacral dorsal commissural nucleus). AADC antibody was from rabbit. Scale bar in **(C)**, valid for **(A–D)**, 200 μm; in **(G)**, valid for **(E–K)**, 50 μm.

**FIGURE 7:**
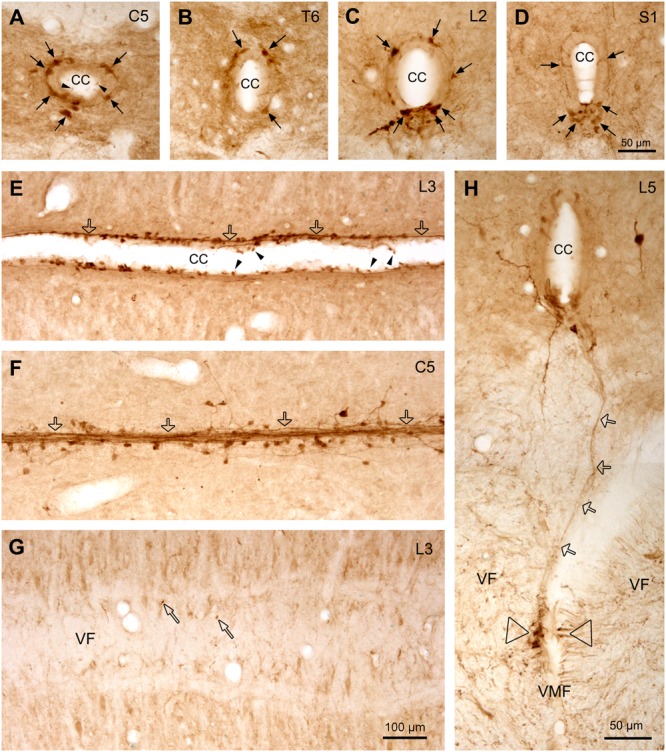
Aromatic L-amino acid decarboxylase cells and fibers/fiber bundles around the CC. **(A–D)** AADC cells around the CC from C **(A)**, T **(B)**, L **(C)**, and S **(D)** segments illustrated in transverse sections. Dorsal side is upward for all the sections. It is clear that at C and T spinal levels the AADC cells were distributed evenly around the CC in different directions, whereas at the L and S levels, especially at S level, the AADC cells were mainly located ventral to the CC although a small number of the cells could also be observed in other parts (arrows). **(E,F)** AADC cells and fibers/fiber bundles lateral to the CC **(E)** and just at the bottom of the CC **(F)** seen in horizontal sections. It seems that the fibers arising from the AADC cells joined together to form fiber bundles (hollow arrows) which run longitudinally along the CC, yielding garlic braid-like chains. **(G)** Image from a horizontal section just below the CC showing that only a few AADC-IR puncta are apparent (narrow hollow arrows) in VF, indicating that most fibers from the AADC cells around the CC do not project toward the VMF. **(H)** Image from a transverse section showing a AADC-IR fiber/fiber bundle (hollow arrows) ran all the way from the ventral side of the CC to the left side of the VMF, where a cluster of AADC-immunoreactive fibers is apparent (hollow triangles). This fiber/bundle might be originated from an AADC cell(s) around the CC but it is hard to pinpoint its originating cell body in this section. In some sections, e.g., in **(A)** and **(E)**, clear swellings at one end of the AADC cell processes could be seen to protrude into the lumen of the CC (arrowheads). The spinal segment level is indicated at upper right corner in each panel. AADC antibody was from rabbit. Scale bar in **(D)**, valid for **(A–D)**, and in **(H)**, 50 μm; in **(G)**, valid for **(E–G)**, 100 μm.

**FIGURE 8:**
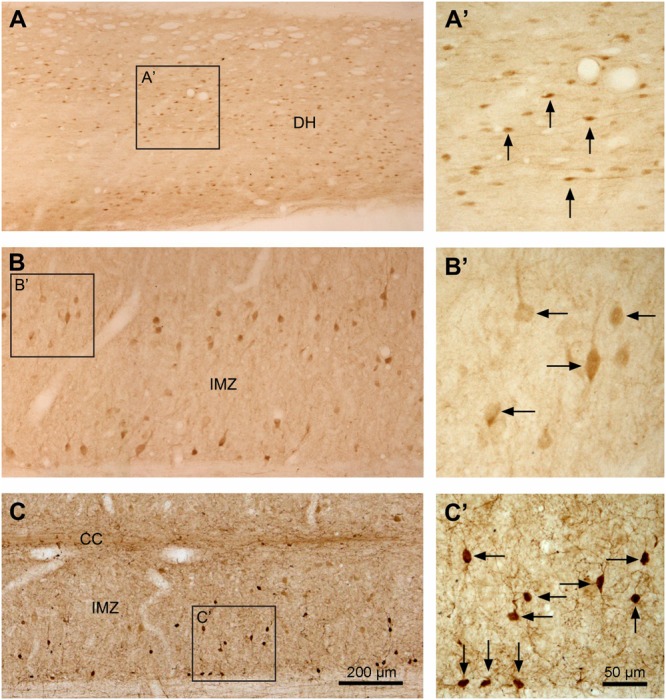
Aromatic L-amino acid decarboxylase cell distribution in different regions of the spinal gray matter illustrated in horizontal sections. **(A–C)** Photomicrographs of low magnification showing the AADC cells in the superficial DH in L5 segment **(A)**, in the IMZ in the same segment **(B)**, and IMZ in S4 segment **(C)**. **(A’–C’)** are the enlargements of the squared regions in **(A–C)**, respectively. Note that the small fusiform- or spindle-shaped AADC cells in the superficial DH with their long axes oriented in the rostrocaudal dimension **(A,A’)**. In the IMZ in the L segment the AADC cells were larger and most of them had their long axes oriented mediolaterally **(B,B’)**. The AADC cells in the IMZ in the S segment were smaller and the long axes were oriented in different directions **(C,C’)**. Arrows indicate the representative AADC cells in different regions. Medial side is upward for **(A,B)**. AADC antibody was from rabbit. Scale bar in **(C)**, valid for **(A–C)**, 200 μm; in **(C)’**, valid for **(A’–C’)**, 50 μm.

**FIGURE 9:**
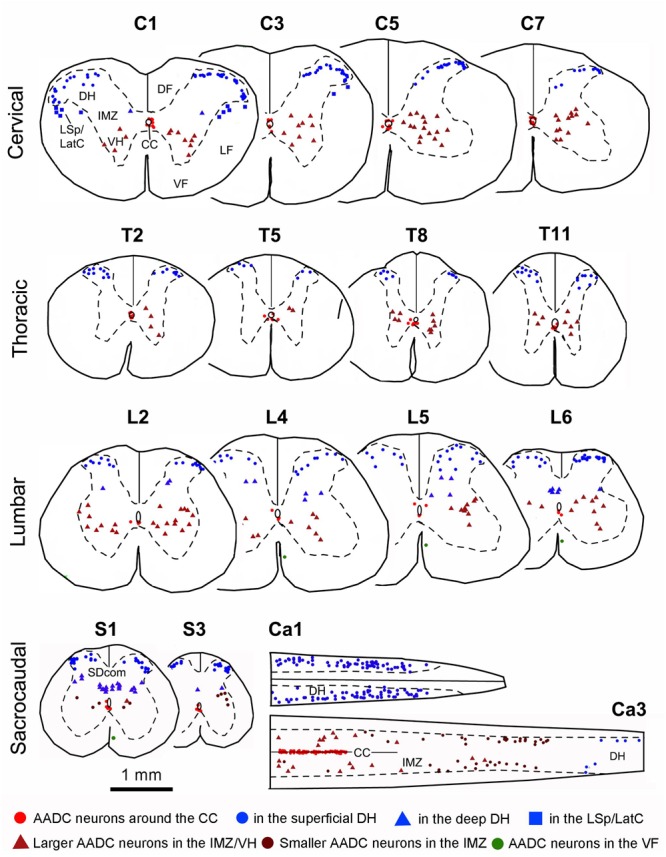
Plots of AADC cells in different segments of the spinal cord. Shown are the representative sections from C, T, L, S, and Ca segments from one rat. The segment level is indicated above each section. Sections from C1 to S3 levels were transverse and the two sections from Ca1 to Ca3 levels were horizontal. Of the horizontal sections, the top one is through the superficial DH and the bottom one is partly through the CC and partly the IMZ. DF, dorsal funiculus; LatC, lateral cervical nucleus; LSp, lateral spinal nucleus; LF, lateral funiculus; SDcom, sacral dorsal commissural nucleus; VF, ventral funiculus; VH, ventral horn. Because AADC neurons in the VF were not plotted, for the purpose of illustration, they were manually placed in L4, L5, and S1 sections (green dots) (please refer to **Figure 11**). Spinal sections used for plotting were immunostained with rabbit AADC antibody. Bar below sections S1 and S3, valid for all panels, 1 mm.

**FIGURE 10:**
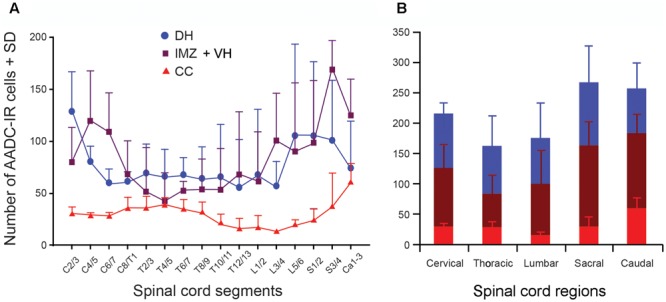
Histograms showing the density of the AADC cells in different spinal segments. **(A)** AADC cell (neuron) distribution in the spinal gray matter. The density of AADC cells (number/mm^3^ spinal tissue) in three different locations – the DH, IMZ plus ventral horn (IMZ + VH), and around the CC – were plotted in relation to the spinal segments. For the AADC cells in the DH the rostral cervical segments (C2/3) contained the highest density of the cells, and the caudal thoracic segments (T12/13) contained the lowest density of the cells. For the AADC cells in the IMZ + VH the caudal sacral segments (S3/4) contained the highest density, and the rostral thoracic segments (T4/5) contained the lowest density of the cells. For the AADC cells around the CC the highest densities were found in the caudal spinal cord and the lowest densities in mid-lumbar segments (L3/4). **(B)** Summarized data from **(A)** illustrating the AADC cell distribution in five spinal regions.

**FIGURE 11:**
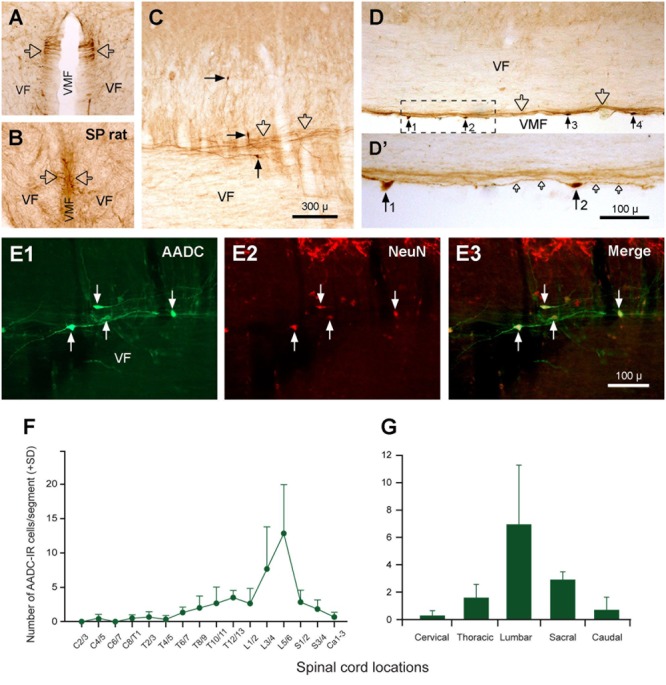
Aromatic L-amino acid decarboxylase cells (neurons) and fibers in the VF along the VMF. **(A)** A transverse section at the first sacral level from a normal rat showing the AADC fibers in the VF close to VMF (hollow arrows). **(B)** A transverse section at the fourth sacral level from a 70-day S2 spinalized (SP) rat showing that the AADC fibers in this location persisted after spinal cord transection (hollow arrows). **(C,D)** AADC neurons (black arrows) and fibers (hollow arrows) in the VF close to the VMF in a parasagittal **(C)** and a horizontal **(D)** section. **(D’)** The enlargement of the area delimited by the dashed line in **(D)**. Four AADC cells (No. 1–4) were seen in **(D)** and it seems that all of the cells gave rise to fibers from their two ends and projected along the VMF (small hollow arrows in **D’**). **(E1–E3)** AADC cells in the VF were NeuN-IR, demonstrating their neuronal property. **(F,G)** Histograms showing the relative abundance of the AADC neurons in VF in relation to the spinal segments **(F)** and regions **(G)** (same format as **Figure 10**). It can be seen that the lumbar region, especially L5/6 segments, contained the highest density of the cells, the cervical region contained the lowest density of the cells, and the other regions were in between. AADC antibody was from rabbit. Scale bars in **(C)**, valid for **(A–D)**, 300 μm; in **(D’)**, 100 μm; in **(E3)**, valid for **(E1–E3)**, 100 μm.

L-Amino acid decarboxylase-immunoreactive cells were found in every segment of the spinal cord from C through Ca levels. They were found to be located both in the spinal gray matter and white matter (**Figures 2–11**). In the gray matter AADC-IR cells were seen in the region around the central canal, in the dorsal horn, intermediate zone, and ventral horn. To identify the phenotype of AADC cells in different regions of the spinal cord we have double-stained spinal sections with AADC and NeuN, doublecortin, ChAT, VAChT, GFAP, APC, Iba1, or BLBP antibodies. The results showed that in the spinal gray matter AADC cells were NeuN-immunopositive (**Figures 2A1–C3**), demonstrating their neuronal identity. However, the AADC cells around the central canal displayed a weaker NeuN labeling than the cells in the dorsal horn or the intermediate zone/ventral horn (cf. **Figures 2A1–A3,B1–C3**). In contrast, AADC cells around the central canal displayed a stronger doublecortin labeling than other regions (**Figures 3A1–C3**), indicating the immature neuronal property of the AADC cells around the central canal. AADC cells were not ChAT-IR in any of the regions in the gray matter (**Figures 4A1–C3**). In addition, AADC was not expressed in motoneurons labeled with VAChT antibody in the ventral horn (**Figures 4D1–D3**). These results indicate that AADC neurons in the spinal cord gray matter are not cholinergic.

In the white matter AADC-IR cells were found in different funiculi (**Figures 6A–E**). These cells were not double-labeled with NeuN (data not shown), indicating their glial nature. Double-staining with AADC and glial marker antibodies showed that in the white matter the AADC cells were double-labeled with APC or BLBP but not with GFAP or Iba1 (**Figure 5**), indicating that AADC was expressed in oligodendrocytes and/or radial glial cells but not in astroglial, or microglial cells. From **Figure 5** it is obvious that only a small portion of glial cells in the white matter expressed AADC and almost all of these cells were immunolabeled with APC or BLBP (**Figures 5C1–D3**); thus, even without confirmation by immunostaining with AADC, APC, and BLBP triple antibodies, it is plausible that a subset of glial cells which expressed AADC also expressed APC and BLBP. There were no AADC-IR cells double-labeled with GFAP, APC, or Iba1 in the gray matter (data not shown).

Although AADC was expressed in glial cells our emphasis in the following sections is on describing the distribution of AADC neurons in different regions of the spinal cord.

The general distribution pattern of the AADC neurons in the gray matter was similar across different segments of the spinal cord although there were some notable differences. According to their locations AADC neurons could be divided into three groups: those around the central canal (lamina X), those in the dorsal horn (laminae I–VI), and those in the intermediate zone/ventral horn (laminae VII–IX) (**Figures 6–10**). We group together AADC neurons in the intermediate zone and the ventral horn because of their similar morphological characteristics. AADC neurons around the central canal were usually small (ca. 7 × 7∼15 μm × 15 μm in short and long dimensions) and densely immunolabeled (**Figures 6, 7**). Their cell bodies, which are usually round, oval or wedge-shaped, typically lie in the subependymal region. These cells have a process that extends through the ependymal layer and ends in a swelling that protrudes into the central canal (**Figures 7A,E**). Thus, they appear to belong to the family of cerebrospinal fluid (CSF)-contacting cells (Vigh et al., 1977). Such cells were observed in every spinal segment from the C to Ca part, and even in the filum terminale (not shown). In the C and T segments they were distributed rather evenly around the central canal (**Figures 7A,B**). More caudally, their locations gradually shifted in a ventral direction so that in the L and S + Ca segments they were mostly located along the ventral aspect of the canal, with occasional cells occurring dorsally and laterally (**Figures 7C,D**).

Aromatic L-amino acid decarboxylase neurons in the dorsal horn could be generally divided into two groups: one in the superficial dorsal horn (laminae I–III) and one in the deep dorsal horn (laminae IV–VI). In the superficial dorsal horn they were predominantly located in lamina II, especially in its inner layer, although occasionally they could also be observed in laminae I and III (**Figures 6A–D,G,K, 8A,A’**). AADC neurons in the superficial dorsal horn were small (ca. 5 × 8∼7 μm × 15 μm) and moderately to densely immunolabeled. When observed in transverse sections their cell bodies appear to be round (**Figure 6G**); however, in horizontal sections almost all of them were oval or fusiform-shaped with their long axes running parallel to the longitudinal axis of the spinal cord (**Figures 8A,A**’). In the deep dorsal horn the AADC neurons were very sparse. Numerous AADC neurons were observed in two nuclei, which either belong to or are at least associated with the dorsal horn. The first of these nuclei is the lateral cervical nucleus (probably also includes lateral spinal nucleus) which is a small neuronal group located in the ventrolateral part of the dorsal horn in the upper C segments (**Figure 6A**). AADC neurons in this nucleus were larger in size (ca. 10 × 20∼20 μm × 30 μm), mostly oval or triangular in shape and moderately to densely immunolabeled (**Figures 6A,I**). The second is the sacral dorsal commissural nucleus located in a region above the central canal from L6 to S4 spinal segments (Heise and Kayalioglu, 2009) (**Figures 6D,K**). The size and the shape of the AADC cells in this nucleus were generally similar as those in the lateral cervical nucleus with the difference that the intensity of the labeling was less (cf. **Figures 6I,K**). Differences in size, labeling intensity, and localization suggest the division of AADC neurons in the intermediate zone/ventral horn into two subtypes: a group containing larger (10 × 15∼20 μm × 30 μm), weakly immunolabeled cells and a group containing smaller (8 × 12∼12 μm × 15 μm), intensely immunolabeled cells (**Figures 6, 8**). The larger cells were usually found in C to L spinal segments (**Figures 6A–C,H, 8B,B’**) although smaller cells could also occasionally be seen in the T and L segments in the area lateral to the central canal (arrow in **Figure 6J**). The larger cells were more abundant in the C and L segments that constitute the spinal cord enlargements (**Figures 6C, 8B,B’**). The larger AADC neurons were mostly located in the intermediate zone although occasionally they could also be seen in the ventral horn (**Figures 4C1–D3**). However, because they were not ChAT- or VAChT-IR, these calls are not motoneurons. Most of these cells were oval-shaped although some of them had a triangular or multipolar shape with their long axes usually oriented mediolaterally (**Figures 8B,B’**). At the sacral and caudal spinal level, where most of the cells were located in the lateral region (**Figures 6D,F, 8C,C’**), smaller AADC neurons were observed in the intermediate zone. These smaller neurons usually had round or oval cell bodies and their long axes (or long processes) were oriented in different directions although the majority showed a mediolateral orientation.

We have observed sparsely distributed AADC-IR cells in the ventral funiculus along the ventral median fissure. These cells were easily seen in horizontal or parasagittal sections (**Figures 11C–E3**) and were usually small (5 × 10∼10 μm × 20 μm), fusiform-shaped, with two poles that extended fibers in the rostrocaudal dimension. To confirm that these cells were neurons (and not large buttons of fibers of en-passage or growth cones of nerve fibers) AADC and NeuN double immunostaining was performed. The results showed that these cells were NeuN-immunopositive (**Figures 11E1–E3**), demonstrating that these cells are neurons located in the ventral funiculus. These cells were mostly seen in the L segments followed by S and T segments, with only a few in the C and Ca segments (**Figures 11F,G**, see further description below). As discussed later these cells are most likely the origin of the AADC fibers running along the ventral median fissure.

### Segmental Distribution of AADC-IR Cells in the Spinal Cord – A Quantitative Analysis

As stated above we have noticed that there appear to be segmental differences in AADC cell distribution. In order to obtain a better picture of their distribution in different spinal segments, we have plotted AADC cells in three rats and made a quantitative analysis of the AADC cell distribution in relation to the spinal cord segments (**Figures 9, 10**). In addition, horizontal sections from three rats were used to manually count the AADC neurons along the ventral median fissure (**Figures 11E1–G**). Different symbols were used to represent different subtypes of AADC-IR cells based on location, shape, size, and immunolabeling intensity (**Figure 9**). To simplify the quantification, AADC neurons in the gray matter were divided into three groups according to their location: those around the central canal, those in the dorsal horn, and those in the intermediate zone/ventral horn. Neurons in the dorsal horn and the intermediate zone/ventral horn were further divided into several different subgroups according to differences in their morphology and immunolabeling intensity. As the spinal sections from adjacent segments usually showed a similar pattern of AADC immunolabeling we have made a histogram showing AADC cell distribution along the rostrocaudal axis by averaging the data from two adjacent segments. The C1 segment was completely sectioned and plotted only in one rat so we decided to exclude this segment from further analysis. Thus, altogether we present data of 16 spinal segments from the analyzed rats (data from the three Ca segments were pooled together because they were all cut horizontally so that it is difficult to distinguish one Ca segment from another) (**Figures 10A, 11F**). We offer an alternative presentation of these data in **Figures 10B, 11G**, where we pooled data according to the five major spinal regions: C, T, L, sacral, and caudal. We should stress that we do not intend to make an analysis of the absolute number of AADC cells; rather, our aim is to analyze differences in their distribution in different parts of the spinal cord. Accordingly, we did not attempt to adjust for shrinkage that occurred during tissue processing.

As shown in **Figure 10**, as a whole, excluding the AADC neurons along the ventral median fissure, the C through Ca levels of the spinal cords of three rats contained, on average, 186.8 ± 68.2/mm^3^ AADC neurons. The largest population of AADC neurons was in the intermediate zone and ventral horn (82.9 ± 29.1/mm^3^; about 70% in the intermediate zone and 30% in the ventral horn) followed by that in the dorsal horn (76.2 ± 35.0/mm^3^). The smallest population of AADC neurons was in the region around the central canal (27.6 ± 7.0/mm^3^). When comparing the AADC neurons in the above three regions, the density of AADC neurons was highest in the S segments (267.1 ± 110.0/mm^3^), followed by the Ca (257.4 ± 80.7/mm^3^), C (216.5 ± 46.2/mm^3^), L (175.8 ± 114.5/mm^3^), and T segments (163.3 ± 82.6/mm^3^) (**Figure 10B**). However, one-way ANOVA analysis indicated that there was not a statistically significant difference in the density of AADC neurons in different regions (*F* = 0.80, *P* = 0.55). When analyzed separately, the Ca segments contained the highest density of AADC neurons around the central canal (69.8 ± 18.3/mm^3^), followed by the C (30.2 ± 5.3/mm^3^), S (30.0 ± 18.1/mm^3^), T (29.0 ± 8.0/mm^3^), and L segments (15.6 ± 4.9/mm^3^). One-way ANOVA analysis indicated that there was a statistically significant difference between the different regions (*F* = 5.12, *P* < 0.05). A subsequent Tukey’s test indicated that this difference was due to the difference between the L and the Ca segments (*q* = 6.15, *P* = 0.01). For the AADC neurons in the dorsal horn the highest density was seen in the Ca segments (103.1 ± 63.1/mm^3^), followed by the C segments (90.2 ± 20.3/mm^3^). The T, L, and Ca segments contained almost the same density of the neurons (78.8 ± 48.7, 76.0 ± 57.8, and 73.3 ± 45.5/mm^3^, respectively). One-way ANOVA analysis indicated that there was not a significant difference between the groups (*F* = 0.188, *P* = 0.94). For AADC neurons in the intermediate zone and ventral horn the highest density was also detected in the S segments (134.3 ± 43.1/mm^3^; intermediate zone: 94.9 ± 47.4/mm^3^; ventral horn: 39.4 ± 5.9/mm^3^), followed by the Ca (124.2 ± 35.5/mm^3^; not easy to separate neurons in the intermediate zone from the ventral horn in horizontal sections), C (97.5 ± 36.5/mm^3^; intermediate zone: 73.1 ± 29.6/mm^3^; ventral horn: 24.4 ± 7.5/mm^3^), L (84.3 ± 52.1/mm^3^; intermediate zone: 73.1 ± 29.6/mm^3^; ventral horn: 24.4 ± 7.5/mm^3^), and T segments (55.5 ± 32.1/mm^3^; intermediate zone: 59.0 ± 39.8/mm^3^; ventral horn: 25.3 ± 15.8/mm^3^). However, one-way ANOVA analysis indicated that there was not a significant difference between the groups (*F* = 1.80, *P* = 0.21).

The AADC neurons in the ventral funiculus along the ventral median fissure were counted manually in three rats and expressed as cell number per spinal segment. As illustrated in **Figures 11F,G**, there were 84.3 ± 12.3 AADC neurons in this region on average per rat. L segments, where 8.3 ± 5.4 cells/segment were seen, harbored the most AADC neurons followed sequentially by S (2.3 ± 0.7), T (1.7 ± 1.2), Ca (0.7 ± 0.9), and C (0.1 ± 0.3) segments. With respect to the distribution of AADC neurons in individual spinal segments, there was an apparent density peak at the L5/6 level, followed by the L3/4 level, which contained 12.8 ± 7.1 and 7.7 ± 6.1 cells/segment, respectively. All other segments contained fewer than 3 cells/segment. One-way ANOVA on Ranks analysis indicated that there was a significant difference among the groups (*H* = 12.75, *P* = 0.013). A subsequent Tukey’s test indicated that there was a significant difference between L and C and between L and Ca segments (*P* < 0.05).

### AADC-IR Fibers in the Spinal Cord

In addition to the AADC-IR cells, there were abundant AADC-IR fibers and varicosities in the spinal gray and white matter. In the gray matter the AADC fibers and varicosities could be seen everywhere from the dorsal horn to the ventral horn. We will not describe their distribution in detail because it is impossible to determine the extent to which they arise from spinal AADC neurons. What we were interested in is where the AADC neurons project in- and outside of the spinal cord. This will naturally be better explored with neural tract tracing experiments which were not included in this study. However, some AADC-IR fiber bundles were observed on the ventral side of the central canal, which looked like they could be arising from the AADC neurons in this region (**Figures 7E,F**). In the horizontal sections these fiber bundles appeared to connect with all the AADC cell bodies along their course, yielding a chain-like structure reminiscent of garlic braids. In the white matter AADC fibers were mainly seen in the lateral funiculus, especially in its dorsal part (**Figures 6A–D**). However, in the ventral funiculus there was a bundle of AADC fibers along each side of the ventral median fissure close to the central canal (**Figures 11A–D**, see also **Figures 6A–D**). These fibers were most likely from the AADC neurons located in the ventral funiculus along the banks of the ventral median fissure (**Figures 11C–E3**) because they persisted even when the spinal cord was transected at upper levels (**Figure 11B**). Some of these fibers might originate from the AADC neurons around the central canal since in some sections scattered AADC fibers could be seen running between the central canal and the ventral median fissure (**Figure 7H**); however, such an origin might be minimal since there were not many transected AADC-IR fibers found ventral to the central canal in horizontal sections (**Figure 7G**).

### AADC Immunolabeling in Blood Vessels in the Spinal Cord

**FIGURE 12:**
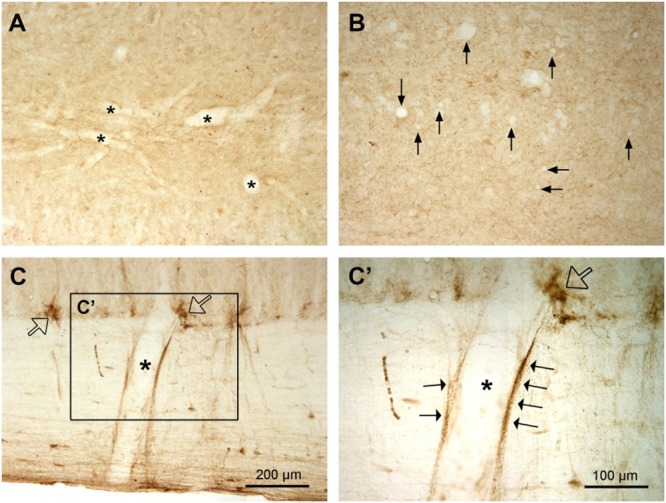
No AADC immunoreactivity in the blood vessels in the spinal cord in normal rats. **(A,B)** AADC immunoreactivity was detected neither in the larger (stars in **A**) nor in the smaller vessels (arrows in **B**). The sections were from a L 6 segment. Note that **(B)** is one-time more amplified than **(A)** (see the scale bars in **C** and **C’**). **(C)** A large vessel (star) in the white matter in a L1 segment seems to be AADC-IR. However, in the enlarged photograph (**C’**, the rectangular area in **C**) the IR components (arrows) seem to come from the associated AADC fiber/varicosity clusters in the intermediate lateral nucleus (hollow arrows in **C** and **C’**). All sections are horizontal sections. **(A,B)** are from regions above the CC. AADC antibody was from rabbit. Scale bar in **(C)**, valid for **(A,C)**, 200 μm; in **(C’)**, valid for **(B)** and **(C’)**, 100 μm.

Since it has been claimed that in the spinal cord a large proportion of AADC enzyme comes from the blood vessels in the spinal cord (Hardebo et al., 1979), it is necessary to describe AADC immunolabeling in the vessels. To our surprise we did not see apparent AADC labeling in the blood vessel walls either in the white or gray matter in normal rats regardless of tissue post-fixation duration. No endothelial cells or pericytes were clearly immunolabeled for AADC (**Figures 12A,B**, see also **Figures 6–8**). However, in the white matter adjacent to the intermediate lateral nucleus in the T and upper L segments the blood vessel walls were indeed associated with AADC-IR components (**Figures 12C,C**’). But upon careful inspection, it appeared that these AADC components were fibers or fiber varicosities that originate in the adjacent intermediate lateral nucleus and run along the surface of the blood vessel wall (arrows in **Figure 12C**’). Two pieces of evidence from our observations support this speculation: first, the AADC-immunolabeled profiles along the vessel wall were not endothelium- or pericyte-like; second, in the segments without the intermediate lateral nucleus there was no such labeling.

## Discussion

Our present study is the first to offer a systematic qualitative and quantitative analysis of AADC cells in the mammalian spinal cord. Our findings disclosed a much wider distribution and a larger number of AADC cells in the rat spinal cord than reported previously (Jaeger et al., 1983, 1984). Further, we found that AADC cells appear to have different morphological properties in different spinal locations. Although the spinal AADC cells in the gray matter seem to be a group of interneurons, some do possess processes that project rostrocaudally for long distances. These characteristics suggest that AADC cells might be subject to further division into several subtypes and that each subtype might exert different functions in different physiological and pathological situations.

### Distribution of AADC Cells in the Spinal Cord

To date, there are only a few studies investigating AADC cell distribution in the mammalian spinal cord (Jaeger et al., 1983; Nagatsu et al., 1988; Li et al., 2014; Wienecke et al., 2014). In general, all studies but ours (Wienecke et al., 2014) have reported the existence of AADC cells only in a region around the central canal in rats or mice, although Li et al. (2014) reported “a novel group of neurons” in the lateral part of the gray matter following S2 spinal transection. Our present study has expanded our previous results by showing that throughout the rat spinal cord AADC cells are not only located in the region around the central canal but also in the dorsal horn and the intermediate zone. The explanation of these differing results may well be that different laboratories have used different AADC antibodies and/or immunohistochemical procedures. AADC cells close to the central canal apparently belong to the category of CSF-contacting neurons found in different animal species (for references see Vigh and Vigh-Teichmann, 1998; Vígh et al., 2004). The AADC neurons in other regions have not yet been characterized.

We are aware of no previous reports of the existence of AADC glial cells in the spinal cord. Yet we have found numerous AADC glial cells in the spinal cord white matter. That most of the AADC glia express BLBP and APC suggests that they are probably immature oligodendrocytes differentiating from radial glial cells. We did not observe AADC expression in mature astrocytes or microglia labeled with GFAP or Iba1. Two studies have reported that cultured astrocytes from rat and mouse neostriatum express AADC (Li et al., 1992; Juorio et al., 1993), but there are no reports as to whether AADC is expressed in brain glial cells with other techniques. This issue definitely deserves to be investigated further.

In the rat spinal cord AADC cells around the central canal have been named D1-cells by Jaeger et al. (1984). The results from our study demonstrated that AADC neurons around the central canal only account for a small proportion of the total number of AADC neurons in the spinal cord (ca. 15%, see section “Results”), and that a large proportion of the AADC neurons are located in the dorsal horn and the intermediate zone/ventral horn. Spinal cord AADC cells not only show different spatial localizations, but, they also display location-dependent differences in morphological and neurochemical characteristics (e.g., labeling with NeuN, doublecortin, and ChAT). This suggests that AADC cells could be further classified into several different subtypes according to their morphological and neurochemical properties. In this study a thorough investigation of neurochemical properties of the AADC cells was not performed, thus we didn’t attempt to make a further classification of these cells.

### Neurochemical Properties of AADC Cells in the Spinal Cord

The primary aim of the present study was to investigate the detailed distribution and general morphology of AADC cells in the spinal cord, rather than their specific neurochemical profiles. Thus, we did not examine whether the AADC cells express monoamine precursors or other essential enzymes to synthesize monoamines. This issue is absolutely worthy of further investigation in order to evaluate how the AADC cells in the spinal cord gain the ability to produce monoamine transmitters following SCI. Although only a small number of AADC cells express TPH and TH, the cells containing these enzymes are located in vicinity of AADC cells (Hou et al., 2016; Zhang, 2016), indicating that 5-HT and DA could be synthesized via a process involving two different cells. As discussed below, this monoamine synthesis mechanism may be critical for the production of monoamines in the spinal cord following SCI.

A number of studies have investigated the neurochemical properties of the CSF-contacting neurons around the central canal in mammals. The results indicate that these neurons are immunoreactive to, among others, glutamic acid decarboxylase (GAD), vasoactive intestinal polypeptide, polysialylated neural cell adhesion molecule, and doublecortin (LaMotte, 1987; Stoeckel et al., 2003; Marichal et al., 2009; Kútna et al., 2014). Recent studies in non-mammalian vertebrates suggest that CSF-contacting neurons in the spinal cord may express acid sensing ion channels (Jalalvand et al., 2016) and PKD2L1 (Böhm et al., 2016) which are sensitive to pH changes and mechanical stimuli, respectively. However, whether AADC cells express these kinds of molecules is largely unknown. We have demonstrated that AADC cells around the central canal are weakly immunolabeled with NeuN, a pattern similar to the CSF-containing neurons reported by Kútna et al. (2014). The combined weak NeuN and stronger doublecortin immunoreactivity suggests that CSF-containing/AADC neurons in this region might be immature neurons that retain the potential for migration and/or differentiation (Marichal et al., 2009; Kútna et al., 2014). Because they are densely immunolabeled with NeuN and immunonegative for doublecortin the AADC neurons in other regions seem to be mature neurons. We did not see AADC expression in ChAT/VAChT-immunopositive cholinergic neurons in any part of the spinal cord. This result is somewhat different from that of Gozal et al. (2014), who have reported AADC expression in ChAT-labeled motoneurons in the ventral horn in the rat spinal cord. It is unclear what causes this difference. However, our published data from S2 spinalized rats also argue against spinal motoneurons expressing AADC because, following 5-HTP application, none of ChAT-labeled neurons expressed 5-HT, while almost 100% of AADC cells expressed 5-HT (see Figure 5 in Wienecke et al., 2014).

### AADC Fibers and Terminals in the Spinal Cord

It is of great functional importance to investigate where the axons of the AADC neurons in the spinal cord project. In our study two main AADC fiber tracts were found in the spinal white matter: one in the dorsolateral funiculus and the other one in the ventral funiculus toward the ventral median fissure (**Figures 6, 11**). As discussed below, the fiber tract in the lateral funiculus might come from the descending monoamine fibers from the brain, whereas the fiber tract in the ventral funiculus might come from the AADC neurons around the central canal. One piece of evidence supporting this assumption comes from Stoeckel et al. (2003), who found thin, unmyelinated axons close to the ventral median fissure expressing the same immunoreactivities as CSF-containing neurons such as GAD, P2X2, and PSA-NCAM. Another piece of evidence supporting this assumption is that the AADC fibers in this region are unaffected by a complete spinal transection (**Figure 11B**). By contrast, AADC fibers in the dorsolateral funiculus disappear following spinal transection, indicating that the AADC fibers in this region come from the structures above the lesion. Moreover, the location of the AADC fibers in the dorsolateral funiculus fits with that of the descending monoamine fibers in the spinal cord (e.g., 5-HT, Basbaum et al., 1988). It needs to be mentioned that Stoeckel et al. (2003) only observed fiber bundles in the ventral funiculus at level T13 and below whereas we have observed the fibers throughout the entire spinal cord (**Figure 6**). What causes the difference is unknown. Perhaps the AADC fibers express different molecular markers in the upper and lower parts of the spinal cord, and/or the AADC fibers in the upper spinal cord come from the supraspinal structures. Since the level of spinal transection in our experiments was always at sacral segments it is presently impossible to resolve this discrepancy.

It would indeed be worthwhile to learn about the projections of the spinal AADC cells, but presently the data on this are very sparse. In *Xenopus* spinal cord Vigh et al. (1977) described that thin tracts of CSF-contacting neurons run across the gray matter to the margin of the ventral funiculus in contact with the basal lamina of the external surface of the spinal cord. LaMotte (1987) described that in the cat axonal terminals from vasoactive intestinal polypeptide CSF-contacting neurons could be traced along the ventral median fissure, the ventral and the ventrolateral surface of the spinal cord. However, it is difficult to judge whether these CSF-contacting neurons are AADC neurons. In addition, the AADC neurons in the dorsal horn and the intermediate zone/ventral horn are newly discovered populations and the data regarding their projection fields are completely absent. From our unpublished results following chronic spinal transection it seems that AADC fibers and varicosities are located in the regions where the cell somata are located, that is, more in the regions around the central canal, the dorsal horn, and the intermediate zone. Using DiI tracing technique Gozal et al. (2014) reported that AADC cells around the central canal project to the ventral funiculus close to the ventral median fissure. However, from our immunohistochemical results these projections are minimal (see **Figures 7G,H**). Thus to further characterize AADC cell functions it is absolutely necessary to make an exclusive study of projection targets of AADC cells in the spinal cord with more advanced neural tracing techniques.

### AADC in Blood Vessels in the Spinal Cord

It is worthwhile to discuss the presence of AADC in the spinal cord blood vessels, as Hardebo et al. (1979) have found that in both normal and spinal-transected rats, more than 70% of AADC activity in the spinal cord is attributable to spinal microvessels. According to their data one may expect that a large number of blood vessels express AADC in normal rat spinal cord. Surprisingly we did not observe AADC immunolabeling in any blood vessels in the spinal cords of normal rats. Li et al. (2014) found that only after spinal cord was transected were the vessels seen to express AADC where 5-HT could also be synthesized when 5-HTP was available. Recently results from the same group (Li et al., 2017) showed that the AADC labeling is located in the pericytes surrounding the capillaries but not in the endothelial cells. Gozal et al. (2014) found a weak AADC vessel labeling in normal rats, which we believe was most likely background labeling. Based on the new finding from Li et al. (2017), the results from Hardebo et al.’s (1979) could be explained as follows: In Hardebo et al.’s experiments the spinal cords were either homogenized or sliced and all the tissues were incubated *in vitro* for a certain period of time. Thus, it would be possible that it is the injury of the spinal tissue *per se* that induces the blood vessels to express AADC – an explanation that would be consistent with Li et al.’s finding.

### Possible Functions of AADC Cells in the Spinal Cord

As AADC is widely distributed in the central nervous system and peripheral tissues, it might be presumed to have different functions in different tissues. Infants with AADC deficiency may have severe developmental delay, weak muscle tone (hypotonia), muscle stiffness, difficulty in moving, and involuntary writhing movements of the limbs (athetosis) (Pons et al., 2004). Animal experiments have shown that AADC is crucial for brain development and motor functions (Shih et al., 2013). Nevertheless, the functions of AADC cells in the spinal cord are very enigmatic considering the fact that in the normal mammalian spinal cord the AADC cells do not contain monoamines.

Given that AADC cells are widely distributed in the spinal cord and that those in different locations often have different morphologies and labeling intensities, it seems unlikely that they serve a single function. Rather, it seems reasonable to assume functional diversity, with the function of each group of AADC cells being determined by its location and the refined morphological and enzymatic profiles of its constituent cells. The AADC cells around the central canal seem to belong to a group of CSF-contacting cells with rather weak, though positive, NeuN-labeling. Therefore, it seems that at least this group of AADC cells may be “immature” and that their function is most likely related to development and plasticity in reaction to different types of challenges, such as neural trauma. Recently some research groups have shown that CSF-contacting neurons in fish could detect mechanical stimulation, pH changes, and regulate locomotion (Böhm et al., 2016; Jalalvand et al., 2016). However, whether these CSF-containing neurons contain AADC is unknown. The other groups of AADC cells in the gray matter appear to be mature neurons and further discussions on their function has to await further investigations on their histochemical profile (including their transmitter contents), their synaptic inputs, and their projection targets. While most of their functions in the normal situation are still under exploration one thing is certain: following SCI, AADC cells increase their ability to produce monoamines from available precursors (Li et al., 2014; Wienecke et al., 2014; Azam et al., 2015; Ren et al., 2016). This capacity is not only related to the development of spasticity (Bedard et al., 1979; Harvey et al., 2006), but also to the recovery of, e.g., locomotion and micturition (Feraboli-Lohnherr et al., 1997; Sławińska et al., 2013; Hou et al., 2016). In such cases the AADC cells could provide lost monoamines by non-synaptic signal transmission through which neural signals/substances from the brain reach the spinal tissue below the lesion via CSF (Vígh et al., 2004). Or alternatively, they could also use monoamine precursors produced from other monoenzymatic cells nearby, such as TPH or TH cells which have been demonstrated to exist in the spinal cord, to synthesize monoamine transmitters (Hou et al., 2016; Zhang, 2016). In such a case the final production of monoamines requires two steps: the first step involving the synthesis of a monoamine precursor in one cell, the second, involving the final production of monoamines in another cell (i.e., AADC cell) (Zhang, 2016). To evaluate such possibilities will need further physiological investigations.

L-Amino acid decarboxylase cells in the spinal cord might also exert other functions by synthesizing trace amines. Imbalance of trace amines in the brain has been demonstrated to be related to a number of neuropsychiatric disorders, including dystonia, Parkinson’s disease, schizophrenia, drug addiction, and mood disorders (Premont et al., 2001; Berry, 2004; Pei et al., 2016). Due to their low concentrations and extremely rapid turnover rates, trace amines are difficult to detect and quantify by conventional immunohistochemistry. However, one of the trace amines, tyramine, was indeed detected with this technique in certain kinds of cells in the rat spinal cord, including AADC cells (Gozal et al., 2014). Although at present no evidence has demonstrated a direct effect of endogenously produced trace amines on spinal neuronal activity, extrinsically applied trace amines, such as tyramine and tryptamine, have been shown to consistently increase motor activity possibly via acting on 5-HT receptors and trace amine-associated receptors, some of which have been shown to be expressed in the spinal cord (Gozal et al., 2014). Considering the large number and wide distribution of AADC cells in the spinal cord and ready supply of trace amine precursors from the blood circulation, it is likely that intrinsically synthesized trace amines from the AADC cells might play transient yet important roles in modulating motor and sensory functions.

## Author Contributions

MZ designed research. L-QR, MC, SG, and MZ performed research. L-QR, MC, YZ, and MZ analyzed data. MZ and HH wrote the paper.

## Conflict of Interest Statement

The authors declare that the research was conducted in the absence of any commercial or financial relationships that could be construed as a potential conflict of interest.
